# A pan-respiratory virus attachment inhibitor with high potency in human airway models and in vivo

**DOI:** 10.1126/sciadv.adv9311

**Published:** 2025-08-01

**Authors:** Gregory Mathez, Paulo Jacob Silva, Vincent Carlen, Charlotte Deloizy, Clémentine Prompt, Anaïs Hubart, Joana Rocha-Pereira, Marie Galloux, Ronan Le Goffic, Francesco Stellacci, Valeria Cagno

**Affiliations:** ^1^ Institute of Microbiology, University Hospital of Lausanne, University of Lausanne, 1011 Lausanne, Switzerland.; ^2^ Institute of Materials, École Polytechnique Fédérale de Lausanne, 1015 Lausanne , Switzerland.; ^3^ INRAE, UVSQ, UMR892 VIM, Université Paris-Saclay, 78350 Jouy-en-Josas, France.; ^4^ Department of Microbiology, Immunology and Transplantation, Rega Institute, Virus-Host Interactions & Therapeutic Approaches (VITA) Research Group, KU Leuven, Leuven, Belgium.; ^5^ Bioengineering Institute, École Polytechnique Fédérale de Lausanne, 1015 Lausanne, Switzerland.; ^6^ Global Health Institute, École Polytechnique Fédérale de Lausanne, 1015 Lausanne, Switzerland.

## Abstract

Respiratory viruses can cause severe infections, often leading to hospitalization or death, and pose a major pandemic threat. No broad-spectrum antiviral is currently available. However, most respiratory viruses use sialic acid or heparan sulfates as attachment receptors. Here, we report the identification of a pan-respiratory antiviral strategy based on mimicking both glycans. We synthesized a modified cyclodextrin that simultaneously mimics heparan sulfate and sialic acid. This compound demonstrated broad-spectrum antiviral activity against important human pathogens: parainfluenza virus 3, respiratory syncytial virus, influenza virus H1N1, and severe acute respiratory syndrome coronavirus 2 (SARS-CoV-2). In addition, the compound is active against avian strains of influenza virus, revealing its importance for pandemic preparedness. The compound retains broad-spectrum activity in ex vivo models of respiratory tissues and in vivo against respiratory syncytial virus and influenza virus, using prophylactic and therapeutic strategies. These findings contribute to the development of future treatments and preventive measures for respiratory viral infections.

## Teaser

A nontoxic virucidal compound effectively targets a broad range of human respiratory viruses, including avian influenza strains.

## INTRODUCTION

Respiratory infections remain one of the leading causes of global mortality, driven primarily by RNA viruses from various families, including *Coronaviridae* [e.g., severe acute respiratory syndrome coronavirus 2 (SARS-CoV-2)], *Picornaviridae* (e.g., enterovirus and rhinovirus), *Orthomyxoviridae* [e.g., influenza A virus (IAV)], *Paramyxoviridae* [e.g., human parainfluenza virus (hPIV)], and *Pneumoviridae* [e.g., respiratory syncytial virus (RSV)]. These viruses are transmitted via aerosols and droplets, infecting the respiratory tract, with seasonal outbreaks influenced by social behaviors and environmental conditions (*1*, *2*). Vulnerable populations such as children, the elderly, and immunocompromised individuals are particularly prone to severe lower respiratory tract infections, including bronchiolitis and pneumonia.

Respiratory viruses are under close surveillance for their pandemic potential, linked to their high transmissibility and capacity for rapid mutation, enabling them to adapt to previously unseen environments. In December 2019, SARS-CoV-2 was first identified in China (*3*). As the pandemic unfolded, SARS-CoV-2 evolved into a major pathogen. By late 2020, the FDA approved the first antiviral treatment, followed shortly by the rollout of vaccines—an unprecedented achievement within a year of global spread. More recently, the cross-species transmission of avian IAV H5N1 to mammals has heightened the urgency to contain this pathogen and prevent human infection (*4*, *5*), while other avian influenza strains continue to pose a major zoonotic threat. Despite the threat posed by respiratory viruses, approved treatments are lacking for most of them. Developing broad-spectrum antivirals is critical for future pandemic preparedness and for providing potential therapies. However, finding effective broad-spectrum antivirals relies on conserved targets present in or used by diverse viruses without toxicity for host cells.

Among the inhibitors investigated for antiviral development, those targeting viruses’ attachment to the host cell are of most interest. Viruses use cell surface glycans, such as heparan sulfate, sialic acid, and histo-blood group antigens, to initiate infection (*6*). Antivirals that mimic these sugars can block viral entry by occupying the binding sites, blocking the virus’s natural attachment to the cell surface. Notably, heparin (*7*), carrageenan (*8*–*10*), and nanoparticles (*11*) have demonstrated antiviral activity by mimicking heparan sulfate (*12*). Derivatives of sialic acid were also used in vitro against paramyxovirus (*13*, *14*) and mimics of histo-blood group antigens have shown efficacy against norovirus (*15*). Since many viruses use the same surface glycans, attachment inhibitors are one of the most promising strategies for developing broad-spectrum antivirals.

A major limitation of such antivirals is their reversible interaction with the virus (*6*, *16*). Once administered, the antiviral may dilute within the body (*6*, *16*). For effective viral inhibition, such antivirals must exhibit either a high binding affinity for irreversible attachment or have virucidal properties to neutralize the virus entirely (*6*, *11*, *16*).

Different broad-spectrum virucidal agents have been investigated, including rhodamine and thiobarbituric acid derivatives, which integrate directly into the membranes of enveloped viruses, thereby disrupting their ability to fuse with host cells (*17*, *18*). Although these compounds can interact with any lipid membrane, mammalian cells have repair mechanisms that mitigate potential cytotoxicity, allowing for effective drug concentrations within tolerable limits (*17*, *18*). Other studies have focused on macromolecules that target viral surface proteins (*19*–*21*). For instance, dendrimers can coat viral particles and inhibit their attachment to host cells, although their large size may limit their clinical applicability (*20*, *21*). Alternatively, smaller macromolecules such as cucurbiturils have demonstrated virucidal activity by blocking viral entry and rendering the virus noninfectious, albeit with effective concentrations in the millimolar range (*19*).

In contrast, our previous work demonstrated that modified β-cyclodextrins (CDs) can act as virucidal biocompatible agents by mimicking essential cell surface glycans and inhibiting viruses in the micromolar to nanomolar range. CD modified on their primary face with multiple undecyls terminated with 6′sialyl-*N*-acetyllactosamine (6′SLN) proved to efficiently mimic sialic acid. CD modified with multiple 11-mercapto-1-undecanesulfonate (MUS) mimics heparan sulfates (*22*–*25*), making them promising antiviral agents endowed with a virucidal activity. The only exception is SARS-CoV-2 where CD-MUS showed no virucidal activity (*25*). However, both CDs have some limitations: CD-6′SLN exhibits greater potency in vitro compared to CD-MUS but has so far only been shown to inhibit several strains of human influenza while did not show inhibitory activity against avian strains (*22*–*25*). This is linked to the specificity of avian strains for α2.3 sialic acid glycans, while human strains have preference for α2.6 sialic acid glycans but can also be inhibited by α2.3 expressing molecules (*23*, *26*).

We, therefore, aimed to identify a single strategy mimicking both heparan sulfates and sialic acid harboring a pan-respiratory virus potential. Such broad-spectrum antiviral is highly needed for pandemic preparedness but also for known viruses for which there are no or limited approved antivirals on the market.

Here, we show the results of a single modified CD capable of targeting both heparan sulfate and sialic acid dependent viruses. This compound showed potent efficacy against hPIV3, RSV, human and avian influenza viruses, and SARS-CoV-2. The broad-spectrum activity was confirmed in a human respiratory tract tissue model and in vivo.

## RESULTS

### Antiviral activity of modified CDs against hPIV3

To develop a pan-respiratory antiviral strategy that can be used against clinically relevant human respiratory viruses but also for pandemic preparedness against avian strains of IAV, a combination of previously synthesized CDs was initially considered as an approach to mimic simultaneously sialic acid and heparan sulfate. To investigate the benefit of this approach, hPIV3 was considered as a good candidate since this virus is known to depend on α2,3 sialic acid and heparan sulfate (*14*, *27*–*32*). To test antiviral activity against actively circulating strains, two clinical isolates were used in parallel with a laboratory strain [American Type Culture Collection (ATCC)].

A glycan array assay was used to investigate hPIV3 glycan attachment. After overnight incubation of hPIV viruses at 4°C, the presence of viruses was quantified by the fluorescence intensity of the positive spots in the array. Our data showed that heparin octasaccharide and sialyl-lacto-*N*-neotretatose d (SLNT), an α2,3 sialic acid glycan, were common hits among the three strains tested (fig. S1). To further validate the hPIV3 dependency for sialic acid and heparan sulfates, we tested the capacity of hPIV3 to infect A549 cells deficient in the expression of sialic acid on their surface by solute carrier family 35 member A1 (*SLC35A1*) knockout, or LLCMK2 cells treated with sodium chlorate (fig. S2). Each strain of hPIV3 showed a significant reduction in infectivity for sialic acid knockout cells, comparable to EV-D68 used as a positive control for sialic acid dependency, and for cells with reduced sulfation of heparan sulfate proteoglycans, as for RSV used as a positive control for heparan sulfate dependency.

**Fig. 1.**
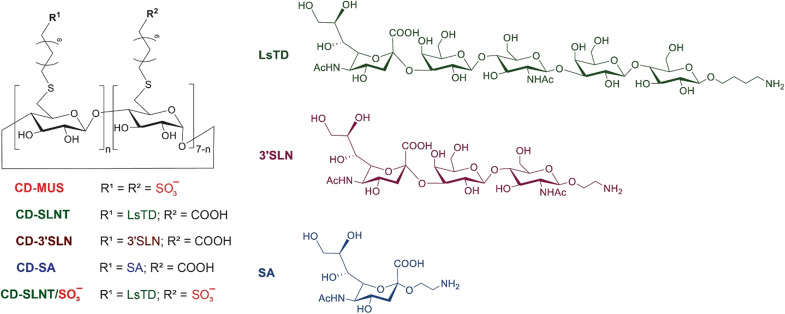
Schematic representation of modified CDs. Schemes of the different CD with the respective ligands. These drawings do not consider the possible configurations the substituents can adopt on the primary face of the CD but only the type of substituents.

To mimic heparan sulfate, CD-MUS (Fig. 1) (*22*) was used. To mimic sialic acid instead, the previously identified CD-6′SLN was not usable since it is not active against avian strains of influenza virus (*23*) and hPIV3 binds to α2,3 sialic acid (fig. S1) (*14*, *28*–*32*). Consequently, CD-3′SLN (Fig. 1) (*23*) was tested as an alternative harboring an α2,3 sialic acid glycan. However, it was not effective against hPIV3, and CD-SA (*33*), a CD harboring the monosaccharide sialic acid, showed low potency against hPIV3 and IAV H5N1 (Fig. 1 and fig. S3A). Therefore, a CD mimicking SLNT (CD-SLNT) was synthesized where the full SLNT glycan is grafted on the primary face of the CD through an undecyl linker (Fig. 1). Both CD-MUS and CD-SLNT showed no toxicity in vitro (fig. S4, A and B).

**Fig. 2.**
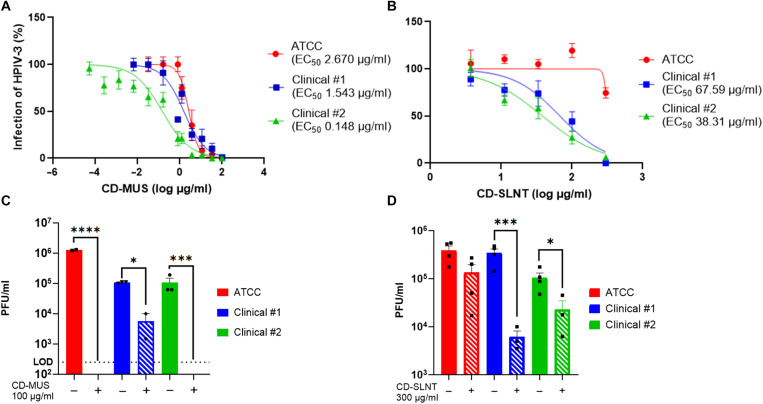
Antiviral activity of CD-MUS and CD-SLNT against hPIV3. Inhibition (**A** and **B**) and virucidal activity (**C** and **D**) of CDs. Inhibition was performed by incubating 1 hour at 37°C laboratory and clinical strains of hPIV3 with CD-MUS (A) or CD-SLNT (B) before infection on cells. Antiviral activity was assessed by plaque assay counted manually. Data represent means ± SEM of two [(A) ATCC], three [(A) clinical #1, (B)], and six [(A) clinical #2] independent experiments. Nonlinear regression with variable Hill slope and constraints for the bottom and top (0 and 100, respectively) were performed to compute EC_50_. (C and D) Virucidal experiments were done by incubating viruses for 1 hour with CD-MUS (100 μg/ml) (C) or 2 hours with CD-SLNT (300 μg/ml) (D) at 37°C. The virus-drug mix was then serially diluted. Viral titer was assessed by plaque assay counted manually. Data represent means ± SEM of two independent (C and D) experiments. Two-tailed *t* tests between untreated and treated conditions were performed. Limit of detection (LOD) *P < 0.0332, ***P < 0.0002, ****P < 0.0001.

To test the antiviral activity of these CDs, the laboratory and clinical strains of hPIV3 were incubated with CD-MUS or CD-SLNT for 1 hour at 37°C before infection. The infectivity was evaluated 3 to 5 days postinfection by plaque assay without the addition of macromolecule after infection. CD-MUS showed an effective concentration inhibiting 50% of virus infection (EC_50_) of 2.7, 1.5, and 0.15 μg/ml (equivalent to 877.78, 496.75, and 48.98 nM) for the laboratory, clinical #1 and #2 strains respectively (Fig. 2A). Next, the number of SLNT glycans on the CD was optimized. Each CD has the potential to have at maximum seven active ligands grafted on the primary face. An equivalence of 1 is synthesized when the number of moles of ligands added to the chemical reaction corresponds to seven times the quantity of moles of CD. CD-SLNT with 0.15 equivalence (i.e., corresponding to a theoretical mean of 1.05 SLNT grafted) showed the best antiviral activity with an EC_50_ of 66 and 38 μg/ml (17.99 and 10.47 μM) for clinical #1 and #2 hPIV3, respectively; however, no effect was found against the laboratory strain (Fig. 2B and fig. S3A). The synthesis of CDs without complete substitution results in mixtures of species, each potentially bearing a different number and spatial arrangement of grafted ligands. Therefore, the CDs grafted with SLNT tested throughout the remainder of the manuscript are mixtures of compounds rather than single, well-defined species.

In addition, the virucidal activity of these materials was assessed by incubating viruses [10^5^ plaque-forming units (PFU)] and a fixed concentration of the drug that showed a full inhibitory activity, 100 μg/ml (32.88 μM) for CD-MUS or 300 μg/ml (81.97 μM) for CD-SLNT, before serially diluting the mix and determining the viral titer by plaque assay. CD-MUS was also shown to be virucidal against the three hPIV3 strains, inducing a complete loss of infectivity of the laboratory and clinical #2 strains, and a significant 1.45 log decrease of infectivity for the clinical #1 hPIV3 isolate (Fig. 2C). CD-SLNT also showed virucidal activity with a 1.81 log and 0.84 log decrease in infectivity of clinical #1 and #2 isolates, respectively (Fig. 2D). As a control, SLNT alone did not show antiviral activity against hPIV3 clinical #2 (fig. S3A).

### Interference between CD-MUS and CD-SLNT

**Fig. 3.**
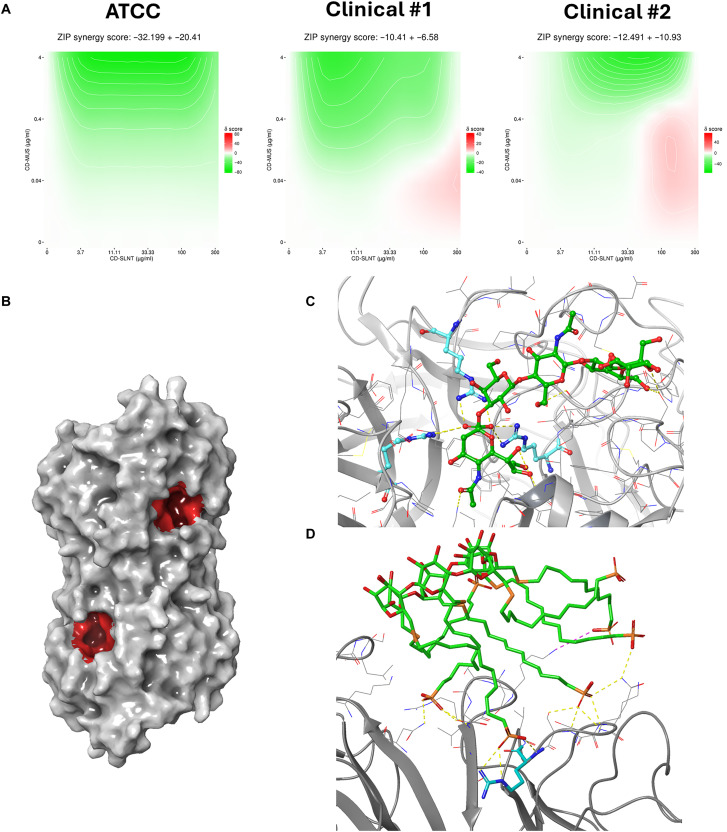
Combination assays using CD-MUS and CD-SLNT. (**A**) CD-MUS and CD-SLNT were mixed at different concentrations and incubated for 1 hour at 37°C with the laboratory or the two clinical strains of hPIV3 before infection of LLCMK2 cells. Inhibition percentages were calculated by plaque assay of two independent experiments and then evaluated with SynergyFinder 3.0 (*34*). The reference algorithms (highest single agent, Loewe, Bliss, and zero interaction potency) were applied. Green, white, and red areas indicate antagonistic, additive, and synergistic areas, respectively. (**B**) The two identical sialic acid–binding pockets (site I) are highlighted in red on the HN of hPIV3 (**C**). SLNT was docked on the boxed sialic acid–binding pocket [PDB 5B2D (*44*)]. (**D**) CD-MUS interactions with the HN protein were simulated by molecular dynamics and showed interaction with this pocket [PDB 4MZA (*47*)]. Ligands carbons are in green and key arginine residues are in cyan.

The combination of CD-MUS and CD-SLNT was then evaluated against hPIV3 infection. CD-MUS and CD-SLNT were mixed at different concentrations and tested against the different strains of hPIV3. Synergy scores were then obtained with SynergyFinder 3.0 (Fig. 3A) (*34*).

For the laboratory hPIV3 strain, results showed a clear antagonism between CD-MUS and CD-SLNT. On the opposite, for hPIV3 clinical isolates, synergy scores are at the limit between antagonism and additivity (i.e., −10). All the different scores to evaluate synergism [highest single agent, Loewe, Bliss, or zero interaction potency (*35*–*40*)], were concordant with the antagonistic effect (table S1).

This antagonist effect was investigated by a computational approach. SLNT was docked on site I of the sialic acid pocket previously described in the hemagglutinin-neuraminidase (HN) protein (*41*–*43*) [Protein Data Bank (PDB) 5B2D (*44*)] (Fig. 3B). The sialic acid was stabilized by a complex of three arginines (Fig. 3C and fig. S5A). SLNT was not possible to dock on site II at the dimer interface (*41*, *45*, *46*). Molecular dynamics of 500 ns were performed with 10 CD-MUS around the HN protein [PDB 4MZA (*47*)]. Most of the amino acids with which CD-MUS interacts by hydrogen bonds, water bridges, or ionic interactions are lysines (30.7%), asparagines (19.5%), threonines (16.5%), and arginines (12.7%) due to their polarity. Although they showed to bind mainly distal locations to the sialic acid–binding site (fig. S5B), interactions were observed in and around the site I sialic acid–binding pocket, including with the arginines interacting with SLNT (Fig. 3D). These data support the antagonism observed experimentally since the binding of one CD could prevent the interaction of the other.

### Pan- activity of the dual-active molecule against respiratory viruses in vitro

To overcome the antagonism of the two CDs, a CD with SLNT and sulfonate as active epitopes on the primary face was synthesized: CD-SLNT/SO_3_^−^ (Fig. 1). CD-SLNT/SO_3_^−^ showed lower EC_50_ on clinical isolates of hPIV3 if compared to CD-SLNT (Table 1). In addition, molecular dynamics support the biological results, confirming that SLNT can bind to the sialic acid pocket while the sulfate can interact with other residues without interference (fig. S5C).

**Table 1.** Antiviral activity of CD-MUS, CD-SLNT, and CD-SLNT/SO_3_^−^. Effective concentration inhibiting 50% of virus infection (EC_50_). Molarities were calculated considering the main synthesized macromolecule.

Virus	CD-MUS (EC_50_) (μg/ml)	CD-SLNT (EC_50_) (μg/ml)	CD-SLNT/SO_3_^−^(EC_50_) (μg/ml)	Virucidal CD-SLNT/SO_3_^−^
hPIV3 ATCC	2.67 (877.78 nM)	>300	56.94 (14,314.23 nM)	Y
hPIV3 Clinical #1†	1.54 (506.28 nM)	65.59 (18,469.74 nM)	0.19 (47.76 nM)	Y
hPIV3 Clinical #2	0.15 (48.98 nM)	38.31 (10,468.64 nM)	0.60 (150.83 nM)	Y
RSV	0.74 (242.62 nM)	>300	2.29 (576.69 nM)	Y
SARS-CoV-2†	5.26 (1729.26 nM)	>300	0.35 (86.73 nM)	Y
IAV H1N1†	6.11 (2007.39 nM)	0.13 (36.32 nM)	0.051 (12.82 nM)	Y
IBV	20.09 (6604.73 nM)	2.61 (711.85 nM)	4.37 (1098.58 nM)	Y
IAV H5N1	54.86 (18,035.61 nM)	0.15 (39.62 nM)	0.55 (138.27 nM)	Y
IAV H7N1	n.a.	n.a.	1.39 (350.44 nM)	n.a.
HSV-2	1.11 (364.92 nM)	>300	20.14 (5063.02 nM)	n.a.

n.a., not assessed.

†, viruses for which CD-SLNT/SO_3_^−^ is more potent than both CD-MUS and CD-SLNT. Y, yes.

This dual-active molecule can mimic simultaneously heparan sulfate and sialic acid and has a broader antiviral activity. CD-SLNT/SO_3_^−^ was therefore tested against a panel of viral pathogens with a multiplicity of infection (MOI) of 0.001 [hPIV3, SARS-CoV-2, and herpes simplex virus 2 (HSV-2)], 0.008 [IAV H1N1, IAV H5N1, and influenza B virus (IBV)], and 0.01 (RSV and IAV H7N1). The macromolecule showed activity against IAV (H1N1), IBV, SARS-CoV-2, RSV, and HSV-2 and also against two different avian strains of IAV (H5N1 and H7N1) (Table 1) in the low micromolar to nanomolar range. To evaluate the variation of EC_50_ in presence of different viral amounts, a dose response assay at different MOIs was performed against IAV H1N1 (fig. S6). The results, evidence, as expected by the mechanism of action, a shift in the EC_50_. However, even at MOI 1, CD-SLNT/SO_3_^−^ has an EC_50_ of 66.62 nM.

Virucidal activity of CD-SLNT/SO_3_^−^ was confirmed against hPIV3, RSV, IAV, and differently than CD-MUS, against SARS-CoV-2 as well. The assay was performed with approximately 10^5^ infectious viruses at a dose of 300 μg/ml (75.42 μM) corresponding to the EC_99_ at a high viral load (Table 1 and figs. S6 and S7). The investigation of virucidal kinetics on IAV and hPIV3 revealed rapid inactivation, even without preincubation for IAV (fig. S8, A and B) or after only 10 min of exposure for hPIV3 (fig. S8C). In addition, the virucidal activity was evaluated in the presence of varying amounts of IAV (fig. S7B), showing—as expected—a more pronounced effect at lower viral loads but still a sustained reduction with a mean of 2.38 log units in infectivity. Moreover, the virucidal activity was validated using an octadecyl rhodamine B chloride (R18) release assay (*33*, *48*), which assesses membrane integrity by measuring the release of a fluorescent dye. As expected, an increase in fluorescence was observed in the presence of 1% Triton X-100 (positive control) and CD-SLNT/ SO_3_^−^, indicating membrane disruption. In contrast, no increase was seen with diethylstilbestrol (DES), which was previously reported to bind hemagglutinin and prevent viral entry without disrupting the membrane (fig. S8D) (*49*).

Characterization of CD-SLNT/SO_3_^−^ by high-performance liquid chromatography coupled with mass spectrometry (HPLC-MS) showed, as expected, a mixture of CDs (fig. S9) with a prevalent species being a CD harboring one SLNT and multiple sulfonate groups. Different batches were synthesized to assess the reproducibility of the synthesis, and the different batches showed consistent activity against IAV H1N1 and RSV (fig. S3, C and E). Furthermore, for viruses dependent on sialic acid (hPIV3 and IAV), an increase of SLNT grafted at the surface of CD-SLNT/SO_3_^−^ reduced the potency of the macromolecule as also shown for CD-SLNT against hPIV3 (fig. S3, B, D, and E), while the effect was not observed for RSV, which does not depend on sialic acid (fig. S3C).

### Resistance to CD-SLNT/SO_3_^−^

**Fig. 4.**
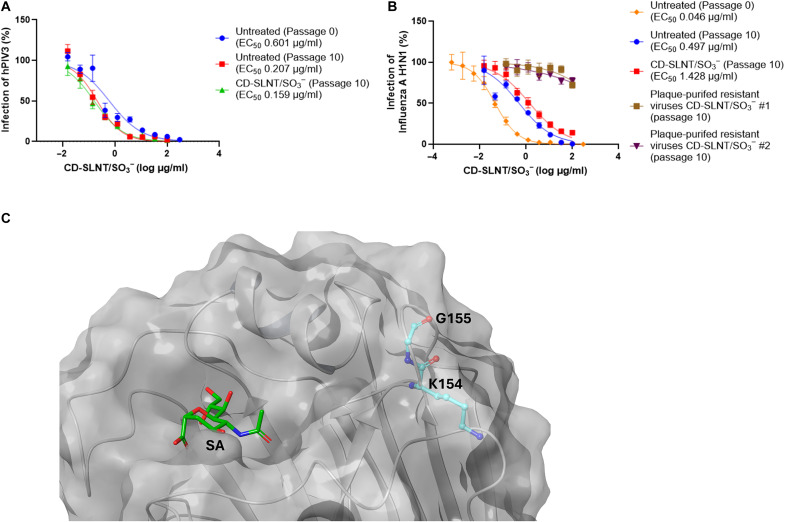
Resistance to CD-SLNT/SO_3_^−^. HPIV3 (**A**) and IAV H1N1 (B) viruses were passaged 10 times in the presence of an increasing concentration of CD-SLNT/SO_3_^−^. At the final passage, the antiviral activity of CD-SLNT/SO_3_^−^ against the untreated and treated viruses was assessed. Resistant plaque-purified IAV H1N1 viruses were obtained (**B**). Data represent means ± SEM of two [(B) plaque-purified resistant CD-SLNT/SO_3_^−^ passage 10], three [(B) untreated passage 10, CD-SLNT/SO_3_^−^ passage 10] or four (A) independent experiments. Each independent experiment with untreated or treated virus at passage 10 was carried out using the two independent virus replicates. Nonlinear regression with variable Hill slope and constraints for the bottom and top (0 and 100, respectively) were performed to compute EC_50_. (**C**) Mutations observed in resistant viruses present in the hemagglutinin protein [PDB 4JTV (*59*)] and sialic acid (SA) at the glycan-binding region are highlighted in cyan and green, respectively.

To investigate the barrier to resistance of CD-SLNT/SO_3_^−^, hPIV3 clinical #2 and IAV H1N1 were passaged 10 times in the presence of an increasing concentration of the macromolecule. After the last passage, the antiviral activity of CD-SLNT/SO_3_^−^ was assessed against the virus passaged 10 times in the presence or absence of the macromolecule. After 10 passages, no resistance of hPIV3 to the macromolecule was detected (Fig. 4A). However, regarding IAV H1N1, the antiviral potency of CD-SLNT/SO_3_^−^ was reduced by 28 times against treated viruses compared to the untreated virus for which the macromolecule already lost 10 times its antiviral activity after 10 passages (Fig. 4B). Two CD-SLNT/SO_3_^−^-resistant IAV variants were purified by plaque assay (Fig. 4B), and the whole genome was sequenced to determine whether specific mutations were present. Several mutations were identified (table S2), two of which, K154E and G155E, are located close to the sialic acid–binding site of the hemagglutinin (Fig. 4C). In the GISAID database, they are observed to be present in less than 1% of the sequenced influenza viruses (table S2).

### Broad-spectrum activity of CD-SLNT/SO_3_^−^ ex vivo

**Fig. 5.**
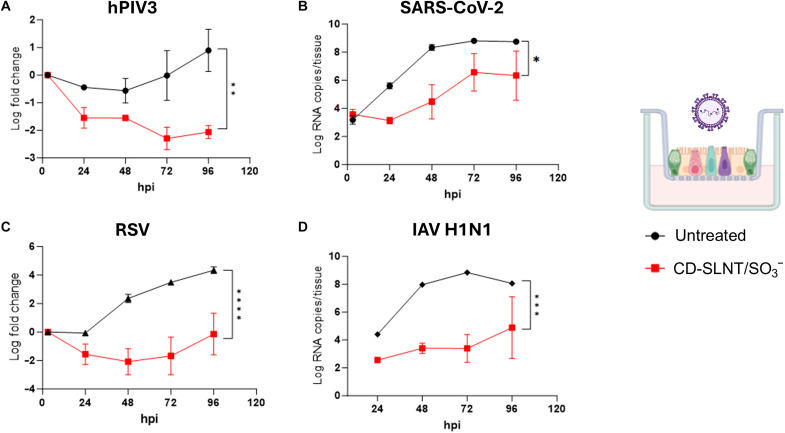
Ex vivo activity of CD-SLNT/SO_3_^−^. HPIV3 (**A**), SARS-CoV-2 (**B**), RSV (**C**), or IAV H1N1 (**D**) were incubated with CD-SLNT/SO_3_^−^ (300 μg/ml) for 1 hour at 37°C. Human upper respiratory tract models [*n* = 2 (A), (C), and (D) or 6 (B) per group] were then infected at 33°C. The inoculum was removed after 3 hours. A daily apical wash was performed, and the level of viruses released was quantified by RT-qPCR. Data represent means ± SEM. Area under the curve followed by a two-tailed *t* test was performed. *P < 0.0332, **P < 0.0021, ***P < 0.0002, ****P < 0.0001. Cartoon created with BioRender.com, Cagno, V. (2025) https://BioRender.com/5w07cwy.

The efficacy of CD-SLNT/SO_3_^−^ was then evaluated in a more relevant model for viral infections: human-derived, pseudostratified airway epithelium from the upper respiratory tract. This model allows the study of clinical strains without cell adaptation and enables the assessment of receptor usage and true viral tropism, as the primary target cells for these viruses are predominantly ciliated cells. HPIV3, SARS-CoV-2, RSV, or IAV H1N1 were preincubated for 1 hour at 37°C with CD-SLNT/SO_3_^−^ (300 μg/ml, 75.42 μM) to assess whether CD-mediated viral inactivation was sufficient to delay viral replication. Human respiratory airways were then infected and maintained at 33°C for 4 days without further addition of the macromolecule. Apical washes were collected every day, and the viruses were quantified by reverse transcription quantitative polymerase chain reaction (RT-qPCR). In all infected tissues treated with CD-SLNT/SO_3_^−^, a significant reduction of apically released viruses was observed (Fig. 5). The infectivity of apically released IAV H1N1 and SARS-CoV-2 viruses was also evaluated at two different time points, showing a significant reduction in infectious virus at 48 hpi for both respiratory viruses (fig. S10, A and B). In addition, an experiment with treatment at the time of infection, without preincubation, was performed with hPIV3, showing a significant reduction in apically released viruses under this condition as well (fig. S10C). CD-SLNT/SO_3_^−^ did not show ex vivo toxicity by the measurement of cell viability or lactate dehydrogenase (LDH) release even with daily addition of the macromolecule (30 μg to 7.54 nmol) (fig. S4, C and D).

### Activity in vivo of CD-SLNT/SO_3_^−^

**Fig. 6.**
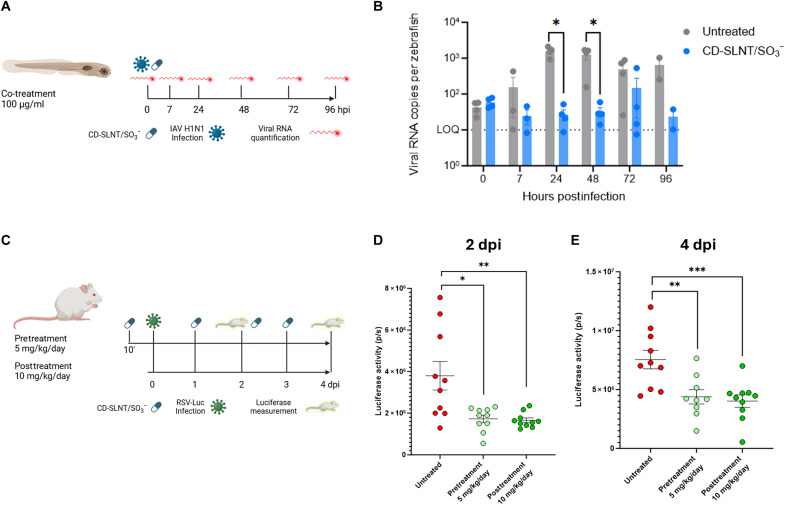
In vivo activity of CD-SLNT/SO_3_^−^. (**A** and **B**) Zebrafish larvae were infected with IAV H1N1. CD-SLNT/SO_3_^−^ was coinjected at the same time at a concentration of 100 μg/ml. At 7 hpi and each day, 10 larvae are lysed for viral RNA quantification by RT-qPCR. Data represent means ± SEM of four independent experiments. (**C** to **E**) Mice (*n* = 10 per group) were infected with RSV. (C) Mice were either treated with CD-SLNT/SO_3_^−^ (5 mg/kg) 10 min before infection (pretreatment) with a daily dose until 4 days postinfection (dpi) or with CD-SLNT/SO_3_^−^ (10 mg/kg) starting 1 dpi (posttreatment). (D and E) RSV replication was assessed by luciferase activity at 2 (D) and 4 (E) dpi. Data represent means ± SEM of a single experiment. Two-tailed Mann-Whitney tests were performed to compare untreated and treated conditions. *P < 0.0332, **P < 0.0021, ***P < 0.0002. Schematic view created with BioRender.com, Cagno, V. (2025) https://BioRender.com/bllixw1.

The antiviral activity of CD-SLNT/SO_3_^−^ was assessed against IAV H1N1 in a zebrafish larvae model. This model has previously been shown to express sialic acid, support IAV infection, and has been used by various research groups to study the host response and pathogenicity of IAV (*50*–*52*). Furthermore, a known antiviral molecule, baloxavir acid, is able to inhibit virus replication, demonstrating that the model is suitable for testing antivirals (fig. S11). IAV H1N1 and CD-SLNT/SO_3_^−^ (100 μg/ml, 25.14 μM) were coinjected into the swim bladder of zebrafish larvae without further addition of the macromolecule (Fig. 6A). A pool of 10 zebrafish were harvested at different time points to quantify the amount of viral RNA. The addition of CD-SLNT/SO_3_^−^ during infection inhibited the replication of IAV H1N1 virus by 1.68 log (Fig. 6B). No signs of toxicity or locomotor impairment (*53*) were observed by the addition of CD-SLNT/SO_3_^−^, in comparison to dimethyl sulfoxide (DMSO)–treated control.

The antiviral activity of CD-SLNT/SO_3_^−^ was further evaluated against RSV in vivo in a mouse model. Mice were infected with an RSV strain harboring a luciferase reporter gene. This infection model has been extensively used to test antiviral efficacy (*11*, *54*). The bioluminescence measured during the experiment has been shown to be a reliable marker of viral load in the lungs and therefore reflects the infection in real time. (*55*). The mice were treated with CD-SLNT/SO_3_^−^ (5 mg/kg per day) 10 min before infection with RSV and daily for 3 days (Fig. 6C). The luciferase activity in the nose and lungs of mice was measured at days 2 and 4 postinfection. CD-SLNT/SO_3_^−^ treatment was shown to inhibit RSV replication at days 2 and 4 postinfection (Fig. 6, D and E). These results were reproduced in an independent experiment (fig. S12A). No loss of weight was observed by the administration of CD-SLNT/SO_3_^−^ in mice (fig. S12B).

Last, the therapeutical activity of CD-SLNT/SO_3_^−^ was also evaluated by treating RSV-infected mice starting 1 day postinfection with CD-SLNT/SO_3_^−^ (10 mg/kg per day, Fig. 6C). In this setting, a comparable global reduction in viral replication was observed (Fig. 6, D and E). For both types of administration, a significant 0.3 log reduction of infection in the lungs of treated mice was observed (fig. S12C) and in the nose of pretreated mice at day 4 postinfection (fig. S12D).

## DISCUSSION

Respiratory viruses continue to pose a dual challenge: a major clinical burden and a persistent pandemic threat. These infections affect individual lives and the global economy, with vulnerable populations—such as children, immunocompromised individuals, and the elderly—often exhibiting insufficient immune responses (*56*, *57*). For these groups, and for viruses without effective vaccines, antivirals remain critically needed. Moreover, the risk of zoonosis is ever-present, as demonstrated by the scrutiny of avian strains of IAV for their zoonotic potential. While vaccination is the preferred strategy for controlling known pathogens, the unpredictable emergence of previously unidentified viruses amplifies the need for broad-spectrum antivirals. Our previous research focused on CD to mimic glycans such as sialic acid and heparan sulfate (*11*, *22*, *24*, *25*, *33*). In this study, we made a breakthrough achievement by designing and evaluating the antiviral activity of a dual-active virucidal macromolecule that mimics both sialic acid and heparan sulfate, resulting in a single macromolecule able to inhibit major respiratory viral pathogens and avian strains of IAV.

An initial combination of the sulfonated CD-MUS and the sialylated CD-SLNT was explored against hPIV3, a virus known to use sialic acid and heparan sulfate as attachment receptors (figs. S1 and S2) (*41*, *58*). Both macromolecules showed antiviral and virucidal activity independently against hPIV3 due to their structural features (Fig. 2) (*22*, *23*). However, the combination of both CDs resulted in antagonism, which was explored with an in silico approach, showing the potential interference of the two macromolecules by binding to the residues close or within the sialic acid–binding region (Fig. 3 and fig. S5, A and B). A similar antagonism is expected as well for other viruses such as IAV H1N1. Lysine, asparagine, threonine, and arginine, the key interacting residues with CD-MUS (fig. S5B), are locally present in the sialic acid–binding region on the hemagglutinin of IAV (*59*–*61*) and could interact with the glycans mediating the interference.

We therefore designed and synthesized a dual-active CD that incorporates SLNT and sulfonate groups on its primary face, referred to as CD-SLNT/SO_3_^−^, providing a unique strategy to simultaneously target viruses using either or both receptors (Fig. 1). The macromolecule was inhibiting and had virucidal activity against major respiratory viruses (IAV H1N1, IBV, SARS-CoV-2, RSV, and hPIV3) and against two avian strains of IAV (H5N1 and H7N1) (Table 1 and figs. S6 to S8). While CD-SLNT/SO_3_^−^ showed improved antiviral efficacy compared to either CD-MUS or CD-SLNT for each virus tested, the dual-active material demonstrated superior potency against a clinical isolate of hPIV3, IAV H1N1, and SARS-CoV-2 compared to both single-active CD (Table 1), suggesting that the mixture of ligands confers to the material additional chemical properties. The dual-active macromolecule has better efficacy than previous attachment inhibitors. For example, CD-SLNT/SO_3_^−^ showed nanomolar activity against SARS-CoV-2 and IAV H1N1, while other virucidal attachment inhibitors designed with a similar approach but with a different scaffold than CD resulted in a micromolar activity (*62*). In addition, CD-SLNT/SO_3_^−^ has virucidal activity against SARS-CoV-2 in contrast to CD-MUS (fig. S7F) (*25*). Furthermore, the kinetics of virucidal activity observed against IAV suggested a faster inactivation by CD-SLNT/SO_3_^−^ (fig. S8, A and B) than previously developed CD (*22*).

This broad-spectrum efficacy not only establishes CD-SLNT/SO_3_^−^ as a versatile antiviral candidate but also underscores its transformative potential for clinical disease management. By targeting viruses that use either sialic acid or heparan sulfate, this macromolecule could address respiratory viruses not yet tested, such as hPIV1 known to have similar sialic acid glycan affinity than hPIV3 (*28*, *63*), human metapneumovirus, human coronavirus NL63, and SARS-CoV known to use heparan sulfate as attachment receptor (*64*–*67*), IAV H7N9 and virtually all other avian viruses which are reported to bind α2,3 sialic acid (*68*, *69*). Previously unidentified emerging sarbecoviruses or strains of avian IAV could also be inhibited by the CD, making it an important tool for pandemic preparedness. In a clinical setting, such a molecule could revolutionize disease management by providing a single, broad-spectrum treatment option. This would reduce reliance on pathogen-specific antivirals with a narrow spectrum activity. Incorporating CD-SLNT/SO_3_^−^ into therapeutic strategies could facilitate rapid responses during outbreaks and improve outcomes for high-risk populations, including those for whom vaccines are ineffective. While further in vivo testing is needed, the scope of activity achieved here already represents a major breakthrough, underscoring the imperative to continue developing such game-changing molecules.

Despite the promising results, the synthesis of the macromolecule presents challenges as it yields CDs with complex mixtures of substitution levels and ligand positions rather than single, well-defined chemical entities, which represents the main limitation of our study. However, various batches of CD-SLNT/SO_3_^−^ tested against IAV H1N1 and RSV revealed consistent antiviral activity (fig. S3, C and E). Characterization via HPLC-MS indicated that the most abundant species is CD with one single SLNT and multiple sulfonate groups (fig. S9); this is in line as well with the reduced activity of batches with higher degrees of substitution (fig. S3, B, D, and E). Although each batch demonstrates reproducible antiviral activity, this heterogeneity must be considered when interpreting structure-activity relationships. These findings also highlight that developing multivalent materials does not necessarily lead to higher potency.

The barrier to resistance of CD-SLNT/SO_3_^−^ was evaluated against hPIV3 and IAV H1N1. While for hPIV3, no signs of resistance were observed (Fig. 4A), IAV H1N1 revealed a reduction in potency after 10 passages (Fig. 4B). Although cell adaptation might already alter the efficacy of CD-SLNT/SO_3_^−^, additional mutations were identified on two different resistant viruses that may abolish the antiviral activity of the macromolecule (table S2): in the hemagglutinin gene, K154E and G155E mutations are located near the sialic acid–binding site (Fig. 4C), supporting the mechanism of action. Notably, K154E has been shown to decrease potency against CD-SA after passages with a combination of interferon and CD-SA (*33*), while G155E might increase α2,6 sialic acid preference (*70*, *71*). A possible explanation is that SLNT, which terminates with an α2,3 sialic acid, would bind less effectively in the altered sialic acid–binding pocket, as the virus could evolve to use preferentially α2,6 sialic acid. Despite this potential risk of resistance, analysis of more than 35,000 sequences from GISAID indicated that the nucleotide mutations responsible for K154E and G155E were present in less than 1% of sequences (table S2). This suggests that the likelihood of encountering a virus in clinical settings harboring these mutations is low. However, resistance could emerge during infection, and considering this possibility, combination therapy should be considered to delay resistance. CD-SLNT/SO_3_^−^ could be associated with other CDs previously described such as CD-6′SLN or another antiviral, DAS181, which has a host-directed activity by cutting sialic acid and finished a phase 2 clinical study against influenza infection (*23*, *72*).

The activity of CD-SLNT/SO_3_^−^ was verified in more complex models. In ex vivo human respiratory tissues, which proved to be relevant for respiratory viruses (*73*–*76*), the macromolecule reduced apically released hPIV3, RSV, IAV H1N1, and SARS-CoV-2 (Fig. 5 and fig. S10, A and B). To represent a more clinically relevant administration strategy, CD-SLNT/SO_3_^−^ was administered at the time of infection, which also demonstrated effectiveness in reducing apically released hPIV3 (fig. S10C). The broad-spectrum efficacy of CD-SLNT/SO_3_^−^ was then confirmed in zebrafish larvae against IAV H1N1 and in mice against RSV. Both animal models showed to support viral replication and suitable for antiviral assays in vivo (fig. S11) (*11*, *50*–*52*, *54*, *55*). Administration at the time of infection showed efficacy in both models (Fig. 6). In addition, in mice, the use of therapeutic administration against RSV showed similar efficacy further confirming the antiviral activity in vivo (Fig. 6, B and C, and fig. S12).

In conclusion, we have presented a nontoxic virucidal pan-respiratory antiviral with broad-spectrum activity in human airway models and in vivo. All the findings presented support further investigation of CD-SLNT/SO_3_^−^ as a potential treatment for human respiratory viral infections effective as well for avian strains of IAV, which could be formulated, for example, as an inhalable dry powder (*77*), with a high likelihood of being effective against previously unidentified emerging respiratory viruses, making it key for pandemic preparedness.

## MATERIALS AND METHODS

### Cells and viruses

LLCMK2 (ATCC CCL-7), A549 (ATCC CRM-CCL-185), MDCK-Siat, A549 SLC35A1 KO, and Vero E6 (ATCC CRL-1586) were given by C. Tapparel, M. Schmolke, and G. Kobinger. The cells were maintained in Dulbecco’s modified Eagle’s medium (DMEM) (Gibco) with 10% fetal bovine serum (FBS) (Pan Biotech) and 1% penicillin-streptomycin (Gibco) at 37°C and 5% CO_2_.

HPIV3 was purchased on ATCC, and two clinical samples were isolated from clinical specimens. IAV [A/Vietnam/1203/2004 (H5N1), and A/Netherlands/602/2009 (H1N1)] were provided by M. Schmolken and IBV (B/Washington/02/2019) by the Sentinella Center for Influenza Virus at the University Hospital of Geneva. Enterovirus D68 (EV-D68) was given by C. Tapparel. SARS-CoV-2 BA.1 Omicron was isolated from a clinical sample (*78*). RSV–green fluorescent protein (GFP) was obtained from (*79*).

### Glycan array

The glycan array was purchased from ZBiotech (ref. 10601-8S) harboring 100 different glycans at its surface. The array was initially blocked for 1 hour at room temperature with phosphate-buffered saline (PBS) (Bischel), 1% bovine serum albumin (BSA) (AppliChem), and 0.05% Tween-20 (Sigma-Aldrich) (blocking solution). Nondiluted viral stock of hPIV3 (laboratory and clinical isolates) [ranging from 1.69 to 4.79 10^4^ focus-forming unit (ffu)/ml] was added to two subarrays for each strain overnight at 4°C. The array was then washed three times with PBS, 0.05% Tween 20 (wash solution) before fixation with PBS, and 4% formaldehyde. The glycan array was incubated in the presence of anti-parainfluenza (1:10) (Light Diagnostics) in blocking solution for 1 hour at 37°C and then washed three times for 5 min each. The array was then incubated with anti-mouse Alexa Fluor 488 (1:1500) (Invitrogen) in the blocking solution for 1 hour at 37°C. After three washes of 5 min, the array was disassembled and immersed in the wash solution for 10 min. Deionized water was used for the last wash for 5 min before drying the glycan array. The array was visualized with Leica DMi 8, and the intensities of the different positive spots were quantified with ImageJ.

### Sialic acid knockout cells’ infection assay

A549 and A549 SLC35A1 KO cells (16,000 cells per well) were seeded in a 96-well plate. Cells were infected with hPIV3 or EV-D68 with a 1:10 dilution of the viral stocks (ranging from 1.69 to 4.79 10^4^ ffu/ml for hPIV3 and 1.48 10^7^ ffu/ml for EV-D68) in DMEM, N-tosyl-L-phenylalanine chloromethyl ketone (TPCK trypsin) (200 ng/ml; Sigma-Aldrich) or DMEM and 2.5% FBS, respectively. After 1 hour at 37°C, the infected cells were incubated in the infection medium for 1 day at the same temperature. After fixation with pure methanol, the cells were incubated in the presence of PBS, 1% BSA, and 0.05% Tween-20 for 20 min at room temperature. The cells were then incubated with either anti-parainfluenza (1:10) or anti-enterovirus (1:500) in PBS, 1% BSA, and 0.05% Tween-20 for 1 hour at 37°C, followed by three washes with PBS, 0.05% Tween 20. The cells were then incubated with PBS, 1% BSA, and 0.05% Tween-20, and anti-mouse horseradish peroxidase (HRP) (1:1000) (Cell Signaling Technology) for 1 hour at 37°C. After three washes, tetramethyl benzidine (Invitrogen) was added to react with HRP, which was stopped by the addition of HCl after enough differentiation of the signal compared to the background. Absorbance was read at 450 nm with a microplate reader.

### Heparan sulfate dependency experiment

LLCMK2 cells were passed at least five times in the presence of 30 mM sodium chlorate (NaClO_3_) to inhibit sulfation of heparan sulfate proteoglycans. LLCMK2 cells passed similarly without NaClO_3_ were used for the untreated condition.

For hPIV3 infection, the cells were seeded in a 24-well plate (80,000 cells per well). One hundred plaque-forming units (0.001 MOI) of the laboratory or clinical strains were used to infect cells for 1 hour at 37°C in DMEM and TPCK trypsin (200 ng/ml). An overlay of DMEM, 0.5% methylcellulose (MTC) (Sigma-Aldrich), and TPCK trypsin (200 ng/ml) with or without 30 mM NaClO_3_ was added to the cells. The infected cells were incubated for three (laboratory strain) to five (clinical strains) days at 37°C. A solution of water, 20% ethanol, and 0.1% crystal violet (Sigma-Aldrich) was used to fix and stain cells. The plaques were quantified manually.

For RSV-GFP infection, the cells were seeded in a 96-well plate (10,000 cells per well). Two hundred focus-forming units of RSV-GFP (0.02 MOI) was used to infect cells for 1 hour at 37°C in DMEM and 2.5% FBS. DMEM and 2.5% FBS with or without 30 mM NaClO_3_ was added to the cells. Infected cells were incubated for 1 day at 37°C. The cells expressing GFP were then counted manually to measure infectivity.

### Synthesis and characterization of modified CD

CD-MUS, CD-3′SLN, CD-6′SLN, and CD-SA were synthesized similarly to previously published protocols (*22*, *23*, *33*). All other CD used the synthesis of CD with undecyl chains grafted on the primary face with carboxy groups activated with *N*-hydroxysuccinimide (NHS), ready for conjugation with a primary amine (*33*, *77*): for details on the synthesis, consult the Supplementary Materials. In general, the aminated end group was reacted to a specific proportion of NHS-activated CD in DMSO (Sigma-Aldrich) in the presence of triethylamine (TEA) followed directly by dialysis against Milli-Q water using 1.5 or 2 kDa regenerated cellulose membranes (Spectrum Laboratories). The contents of the dialysis bag were then freeze-dried and collected for use.

#### Batches of CD-SLNT

Activated CD was reacted with different ratios of α2,3-sialyl lacto-*N*-neotetraose aminopropylglycoside (Neu5Acα2–3Galβ1–4GlcNAcβ1–3Galβ1–4Glcβ1–4 propylamine SLNT, Asparia Glycomics). For initial tests, batches with 1.2, 0.6, 0.3, and 0.15 equivalents of SLNT per NHS group were synthesized and tested against the viruses. The reactions using only SLNT were done as follows: Using a TEA (Sigma-Aldrich) stock solution of 70 μl of TEA in 500 μl of DMSO (i) 7 mg (0.0021 mmol) of activated CD mixed with 0.3 equivalents of SLNT (4.75 mg, 0.0045 mmol) in 5 ml of DMSO to which 20 μl of the TEA solution was added into a 5-ml round bottom flask and magnetically stirred for 12 hour, followed by dialysis against Milli-Q; (ii) 7 mg (0.0021 mmol) of activated CD mixed with 0.6 equivalents of SLNT (9.5 mg, 0.009 mmol) in 5 ml of DMSO to which 20 μl of the TEA solution was added into a 5-ml round bottom flask and magnetically stirred for 12 hours followed by dialysis against Milli-Q; (iii) 7 mg (0.0021 mmol) of activated CD mixed with 1.2 equivalents of SLNT (19 mg, 0.018 mmol) in 5 ml of DMSO to which 20 μl of the TEA solution was added into a 5-ml round bottom flask and magnetically stirred for 12 hours followed by dialysis against Milli-Q. All batches were freeze-dried and collected as a white powder.

#### CD-SLNT/SO_3_^−^

(i) Batch #1: To 50 mg (0.0150 mmol) of activated CD, 0.15 equivalents of SLNT was used per NHS group (18.7 mg, 0.0177 mmol) with 8 mg (0.064 mmol) of taurine (Sigma-Aldrich) in 10 ml of DMSO. The mixture was magnetically stirred for 12 hours, dialyzed, and freeze-dried. (ii) Batches #2 and #3: Two hundred milligrams of activated CD (0.06 mmol) with 74.8 mg of SLNT (0.07 mmol) and 32 mg of taurine (0.255 mmol) were added to 20 ml of DMSO and stirred magnetically for 12 hours and then dialyzed and freeze-dried. (iii) A batch with two SLNTs (0.28 equivalence) and five taurines per CD—Fifty milligrams of activated CD (0.0150 mmol) with 31.68 mg of SLNT (0.03 mmol) and 9.38 mg of taurine (0.075 mmol) with 50 μl of the TEA solution were stirred magnetically in 10 ml DMSO for 12 hours, dialyzed and freeze-dried.

#### CD-3′SLN/SO_3_^−^

Fifty milligrams of activated CD (0.015 mmol), 21.53 mg of 3′SLN (0.03 mmol), and 9.38 mg of taurine (0.075 mmol) were mixed in 10 ml of DMSO and stirred magnetically for 12 hours, followed by dialysis and freeze-drying.

#### CD-6′SLN/SO_3_^−^

Fifty milligrams of activated CD (0.015 mmol), 21.53 mg of 6″SLN (6′SLNEtNH2) (0.03 mmol), and 9.4 mg of taurine (0.075 mmol) were mixed in 10 ml of DMSO and stirred magnetically for 12 hours, followed by dialysis and freeze-drying.

#### CD-SA/SO_3_^−^

Fifty milligrams of activated CD (0.015 mmol) with 10.5 mg (0.03 mmol) of SAEtNH2 and 3.75 mg of taurine (0.03 mmol) were mixed in 10 ml of DMSO and stirred magnetically for 12 hours, followed by dialysis and freeze-drying.

#### CD-SO_3_^−^

Fifty milligrams of activated CD (0.015 mmol) and 10 mg of taurine (0.08 mmol) were added to 10 ml of DMSO, stirred magnetically for 12 hours, dialyzed and freeze-dried.

The relevant batches were analyzed using HPLC-MS (Agilent 1260 6470 LC/TQ) using a Hilic amide column (Waters, XBridge BEH amide 5 μm, 4.6 mm by 250 mm).

### Dose-response against modified CD

Inhibition assays against hPIV3, influenza virus, SARS-CoV-2, RSV, and HSV-2 were done using a similar protocol based on previously described experiments (*22*–*24*). CDs were serially diluted in DMEM containing for hPIV3 experiments TPCK trypsin (200 ng/ml). Two hundred plaque-forming units or infectious units of the virus were incubated with the CD dilutions for 1 hour at 37°C. The virus/CD mix was then used to infect host cells—LLCMK2, MDCK-Siat, or Vero E6, depending on the virus plated in an appropriate multi-well plate—in duplicate corresponding therefore of an MOI of 0.001 (hPIV3, SARS-CoV-2, and HSV-2), 0.008 (IAV H1N1, IAV H5N1, and IBV), and 0.01 (RSV). After 1 hour of incubation at 37°C, the inoculum was removed, and an overlay or maintenance medium was added (DMEM and 0.5% MTC) (Sigma-Aldrich), TPCK trypsin (200 ng/ml) for hPIV3; DMEM, 2.5% FBS, and 0.5% MTC for HSV-2; DMEM, 2.5% FBS, and 0.6% Avicel GP3515 (SelectChemie) for SARS-CoV-2; DMEM for influenza virus; and DMEM and 2.5% FBS for RSV-GFP).

Infected cells were incubated at 37°C for 1 to 5 days depending on the virus. Fixation and staining were performed using crystal violet for hPIV3 and HSV-2; PBS, 4% formaldehyde and water, 20% ethanol, 0.1% crystal violet for SARS-CoV-2; methanol, immunodetection [Ms X Influenza A (1:2000) (Sigma-Aldrich) or anti-influenza B (1:50) (Light Diagnostics) followed by anti-mouse HRP (1:1000) in PBS, 1% BSA, 0.05% Tween-20] and water, 0.1% tablet of 3,3′-diaminobenzidine, 1.8% H_2_O_2_ for influenza viruses. Plaques for hPIV3, SARS-CoV-2, and HSV-2, or infected cells for influenza viruses and RSV-GFP were quantified manually.

For H7N1, A549 cells (150,000 cells per well) were seeded in 24-well plates. The following day, IAV A/Turkey/Italy/977/1999 (H7N1) modified to express NanoLuc luciferase (*80*) with a 0.01 MOI were incubated for 1 hour with successive dilutions of CD-SLNT/SO_3_^−^ starting from 100 to 0.14 μg/ml in Eagle’s minimum essential medium without FBS supplementation. The cells were then infected with the virus:CD-SLNT/SO_3_^−^ mix for 1 hour. After infection, the inoculum was removed. After 24 hours postinfection, the cell layers were lysed in a passive lysis buffer and lysate was used to quantify NanoLuc luciferase activity as previously described (*80*).

### Virucidal experiment against modified CD

Virucidal assays against hPIV3, IAV H1N1, SARS-CoV-2, and RSV-GFP were done using a similar protocol based on previously described experiments (*22*–*24*). For hPIV3, 10^5^ PFUs of viruses were incubated with CDs (100 or 300 μg/ml) for 1 or 2 hours at 37°C in DMEM and TPCK trypsin (200 ng/ml). For influenza viruses or SARS-CoV-2, between 4 × 10^4^ and 5 × 10^5^ infectious viruses were incubated with CD-SLNT/SO_3_^−^ (300 μg/ml) for 1 hour at 37°C in DMEM. The virus/CD mix was serially diluted in DMEM before infection of host cells (LLCMK2, MDCK-Siat, or Vero E6, depending on the virus plated in an appropriate multi-well plate). After 1 hour of incubation at 37°C, the inoculum was removed, and an overlay or maintenance medium was added [DMEM, 0.5% MTC (Sigma-Aldrich), and TPCK trypsin (200 ng/ml) for hPIV3; DMEM, 2.5% FBS, and 0.5% MTC for HSV-2; DMEM, 2.5% FBS, and 0.6% Avicel GP3515 for SARS-CoV-2; DMEM for influenza virus; and DMEM and 2.5% FBS for RSV-GFP].

The infected cells were incubated at 37°C for 1 to 5 days depending on the virus. Fixation and staining were performed using crystal violet for hPIV3 and HSV-2; PBS, 4% formaldehyde and water, 20% ethanol, and 0.1% crystal violet for SARS-CoV-2; methanol, immunodetection [Ms X influenza A (1:2000) or anti-influenza B (1:50) followed by anti-mouse HRP (1:1000) in PBS, 1% BSA, and 0.05% Tween-20] and water, 0.1% tablet of 3,3′-diaminobenzidine, and 1.8% H_2_O_2_ for influenza viruses. Plaques for hPIV3 and SARS-CoV-2, or infected cells for influenza viruses and RSV-GFP were quantified manually to determine the titer.

### R18 release assay

Octadecyl rhodamine B chloride (R18) (MedChemExpress) was dissolved in ethanol at 5 mM. Two hundred microliters of IAV stock (2.51 × 10^6^ FFU/ml) were incubated with 30 μM of R18 for 1 hour at 37°C in the dark. The non-inserted fluorophore was removed by Zeba Spin Desalting column (7 K MWCO, Thermo Fisher Scientific) using PBS as elution buffer. R18-labeled IAV was diluted in PBS containing 1% Triton X-100, CD-SLNT/SO_3_^−^ (100 μg/ml), or 10 μM of DES. Alternatively, the same dilutions were prepared with unmarked virus. Fluorescence was measured at different time points using Omega plate reader with excitation at 540 nm and emission at 580 nm in Corning 96-well half-area black plates. Wells with only PBS were measured as blank, and their relative light unit (RLU) was subtracted to all measured values.

### Ex vivo human upper respiratory tract experiments

MucilAir from a pool of donors were purchased from Epithelix and were maintained according to the manufacturer’s protocol using their MucilAir medium. The different viruses used for ex vivo experiments (hPIV3, RSV-GFP, IAV H1N1, or SARS-CoV-2) were produced on MucilAir. For hPIV3 and SARS-CoV-2, viral stocks with no amplification or a single passage in cell lines were used.

MucilAir were washed with PBS with calcium and magnesium (PBS++) (Gibco) for 45 min at 37°C. Viruses (5 × 10^4^ to 10^5^ RNA copies) were incubated for 1 hour at 37°C with CD-SLNT/SO_3_^−^ (300 μg/ml) in MucilAir medium. After incubation, viruses with or without CD were added apically to infect MucilAir for 3 hours at 33°C on the apical side. MucilAir were then washed twice with PBS++ and maintained at 33°C. Every day, an apical wash was performed by adding MucilAir medium at the apical side for 20 min at 33°C. A sample of each apical wash was then used for RT-qPCR. IAV H1N1 and SARS-CoV-2 were titered on MDCK-Siat and Vero E6 cells, respectively, followed by immunostaining with Ms. X influenza A (1:2000) or anti–SARS-CoV-2 nucleocapsid (1:1000) (Rockland) and Alexa Fluor 488 (1:2000) (Invitrogen). Infectious units were quantified using ImageXpress Pico (Molecular Devices).

### Toxicity assays in vitro

LLCMK2 cells were seeded in 96 well plate (10,000 cells per well). CDs were serially diluted in DMEM with TPCK trypsin (200 ng/ml) and added to the cells. The cells were incubated at 37°C with the different CDs either for 1 hour before removal and addition of DMEM and TPCK trypsin (200 ng/ml), or 5 days without removal or further addition of CDs the subsequent days. After 5 days, the cells were washed once with DMEM. A solution of 3-(4,5-dimethylthiazol-2-yl)-2,5-diphenyltetrazolium bromide (0.5 mg/ml; Sigma-Aldrich) in DMEM was added for 3 hours at 37°C. The cells were then lysed with DMSO. Absorbance at 570 nm was quantified with a Microplate reader.

### Toxicity assays ex vivo

MucilAir were apically treated daily with 30 μg of CD-SLNT/SO_3_^−^ and maintained at 33°C. Apical washes were done every day with MucilAir medium for 20 min at 33°C. The basal medium was replaced every day. CyQUANT LDH Cytotoxicity Assay (Thermo Fisher Scientific) was used according to the manufacturer’s protocol on apical washes. CellTiter 96 Aqueous Cell Proliferation Assay (3-(4,5-dimethylthiazol-2-yl)-5-(3-carboxymethoxyphenyl)-2-(4-sulfophenyl)-2H-tetrazolium (Promega) was performed on the apical side of MucilAir according to the manufacturer’s protocol.

### Resistance against modified CD

For hPIV3, LLCMK2 were seeded on a six-well plate (300,000 cells per well). HPIV3 clinical #2 was used to infect LLCMK2 in quadruplicates with an MOI of 0.01. After 1 hour at 37°C, the virus inoculum was removed. CD-SLNT/SO_3_^−^ (50 μg/ml) were added onto two infected wells diluted in DMEM with TPCK trypsin (200 ng/ml). After 5 days postinfection, the supernatant was collected and centrifuged at 2000 rpm for 5 min. Viruses were titered on LLCMK2 cells. Subsequent passages were performed with the previous virus (MOI 0.01), and CD concentrations were doubled. At passage 10, CD-SLNT/SO_3_^−^ used was at a concentration of 400 μg/ml. Antiviral activity of CD-SLNT/SO_3_^−^ against viruses produced at that last passage.

For IAV H1N1, MDCK-Siat were seeded on a 24-well plate (80,000 cells per well). IAV H1N1 was used to infect MDCK-Siat in quadruplicates with an MOI of 0.01. After 1 hour at 37°C, the virus inoculum was removed. CD-SLNT/SO_3_^−^ (0.2 μg/ml) were added onto two infected wells diluted in DMEM with TPCK trypsin (1 μg/ml). After 2 days postinfection, the supernatant was collected and centrifuged at 2000 rpm for 5 min. Viruses were titered on MDCK-Siat cells. Subsequent passages were performed with the previous virus (MOI 0.01), and CD concentrations were doubled. At passage 10, CD-SLNT/SO_3_^−^ used was at a concentration of 230.4 μg/ml. Antiviral activity of CD-SLNT/SO_3_^−^ against viruses produced at that last passage.

The resistant influenza A H1N1 viruses were plaque purified by infecting a six-well plate of MDCK-Siat (300,000 cells per well) in the presence of CD-SLNT/SO_3_^−^ (300 μg/ml) diluted in DMEM, 0.5% MTC, and TPCK trypsin (1 μg/ml). After 2 days postinfection, MDCK-Siat plated on 24-well plates (80,000 cells per well) were infected with a plaque in DMEM and TPCK trypsin (1 μg/ml). Viruses were collected after 3 to 4 days, titered, and antiviral activity against CD-SLNT/SO_3_^−^ was evaluated. Two resistant purified viruses were selected, one per treated passage 10 virus replicates.

### RT-PCR and RT-qPCR

For ex vivo experiments, a sample of each apical wash was lysed with TRK lysis, and total RNA was extracted with E.Z.N.A. total RNA extraction (Omega Bio-Tek) according to the manufacturer’s protocol. RT-qPCR was then performed with TaqPath 1-Step RT-qPCR (Thermo Fisher Scientific) according to the manufacturer’s protocol with forward/reverse primers and probe specific for each virus [Vi06439670_s1 (Thermo Fisher Scientific) for hPIV3, 5′- CTCAATTTCCTCACTTCTCCAGTGT-3′/5′-CTTGATTCCTCGGTGTACCTCTGT-3′ and 5′- TCCCATTATGCCTAGGCCAGCAGCA-3′ for RSV-GFP, 5′-GACCRATCCTGTCACCTCTGAC-3′/5′-AGGGCATTYTGGACAAAKCGTCTA-3′ and 5′-TGCAGTCCTCGCTCACTGGGCACG-3′ for IAV H1N1, and 5′ACAGGTACGTTAATAGTTAATAGCGT-3′/5′-ATATTGCAGCAGTACGCACACA-3′ and 5′-ACACTAGCCATCCTTACTGCGCTTCG-3′ for SARS-CoV-2].

For the sequencing of resistant IAV H1N1, the two resistant viruses, the untreated viruses of passage 10, and the original virus were lysed with TRK lysis, and total RNA was extracted with E.Z.N.A. total RNA extraction according to the manufacturer’s protocol. Reverse transcription was performed with SuperScript II (Thermo Fisher Scientific) according to the manufacturer’s protocol using random primers. PCR was then performed with Kapa2G (Sigma-Aldrich) and primers from (*81*). Microsynth performed sequencing using M13 as primer. The analysis of mutation frequency in GISAID database was done as described previously (*49*).

### Docking and molecular dynamics

For SLNT docking, the glycan was created on MOE 2019.0102. PDB 5B2D (*44*) was prepared on Maestro Schrodinger 2021-2 using the protein preparation wizard generating het states with Epik at a pH of 7.0 ± 2.0 and restrained hydrogens only with OPLS4 force field during minimization. SLNT was prepared using LigPrep by generating possible states with Epik at pH 7.4 while retaining specified chiralities. It was then docked with Glide on the PDB 5B2D using extra precision without post-docking minimization and the 3-sialyl lactose present in the PDB 5B2D as a reference for grid generation using a 20- and 56-Å inner and outer cubic boxes, respectively. The results were then inspected visually.

For CD-MUS interaction map, PDB 4MZA (*47*) was prepared on Maestro Schrodinger similarly. CD-MUS were created on MOE and then placed 10 times facing sulfonate groups in front of the protein without touching the protein. Molecular dynamics was then prepared for Desmond simulation by creating an orthorhombic box filled with tip3p water with 0.15 mM NaCl. The molecular dynamics was then run for 500 ns at 300 K and 1.01325 bar. Interactions with sulfonate groups with the protein were extracted and analyzed using Python and Jupyter Notebook. Visualization of the interaction map was done with UCSF ChimeraX 2022-05-29.

For CD-SLNT/SO_3_^−^ molecular dynamic, the docked SLNT was extracted to MOE. While maintaining this structure intact, the rest of the CD-SLNT/SO_3_^−^ ligand was created before incorporating the resulting complete ligand into Maestro Schrodinger. PDB 5B2D with CD-SLNT/SO_3_^−^ having the SLNT groups placed at the sialic acid–binding pocket was used as the initial structure for the molecular dynamic. An orthorhombic box was created filled with tip3p water and 0.15 mM NaCl. Desmond was then used for a molecular dynamic of 200 ns at 300 K and 1.01325 bar.

### Antiviral activity of CD-SLNT/SO_3_^−^ in mice

Female BALB/c mice were purchased at 7 weeks old from the Centre d’Elevage R. Janvier (Le Genest Saint-Isle, France). Mice were housed in negative-pressure isolators in a containment level 2 facility. Food and water were available ad libitum. At 8 weeks of age, the mice were anesthetized with a mixture of ketamine and xylazine (60 and 12 mg/kg, respectively, per mouse) and infected intranasally with RSV-Luc (10^5^ PFU) as previously performed (*11*, *54*, *55*). CD-SLNT/SO_3_^−^ were administered as pretreatment (5 mg/kg per day) intranasally for 10 min before infection followed by a daily dose or as posttreatment (10 mg/kg per day) starting 1 day postinfection with a daily dose under anesthesia of 100% oxygen at a rate of 1.5 to 2 liters min^−1^ mixed with around 4 to 5% (v/v) isoflurane delivered to the anesthesia chamber (XGI-8, Caliper Life Sciences, Hopkinton, MA, USA). Luminescence measurements were performed at 2 and 4 days postinfection 5 min following instillation of 50 μl d-luciferin (30 mg/ml, Perking Elmer). Living Image software (version 4.0, Caliper Life Sciences) was used to measure the luciferase activity with an exposure time of 1 min. Digital false-color photon emission images of mice were generated and show the average radiance (photon per second per square centimeter per steradian). Signals are expressed as total normalized flux (photon per second). The mice were euthanized on day 4 postinfection.

### Zebrafish maintenance

Wild-type AB adult zebrafish were maintained in the aquatic facility of the KU Leuven at a temperature of 28°C under a 14-/10-hour light/dark cycle. Fertilized eggs were collected from adults placed in mating cages and were kept in petri dishes containing Danieau’s solution [1.5 mM Hepes, 17.4 mM NaCl, 0.21 mM KCl, 0.12 mM MgSO_4_, 0.18 mM Ca(NO_3_)_2_, and 0.6 μM methylene blue] at 28°C until the start of the experiments.

### IAV infection, CD-SLNT/SO_3_^−^, and baloxavir treatment of zebrafish larvae

Zebrafish larvae at 4.5 days postfertilization, i.e., once their swimbladder is inflated, they were anesthetized using tricaine (Sigma-Aldrich) and positioned laterally in an agarose mold. Before each experiment, the injection needle was calibrated to ensure precise injection volumes. Microinjections were conducted using an M3301R Manual Micromanipulator (WPI) coupled with a FemtoJet 4i pressure microinjector (Eppendorf) (*53*, *82*, *83*). Each larva received a 3-nl injection into the inflated swimbladder of a mixture containing IAV A/Virginia/ATCC3/2009 H1N1 and CD-SLNT/SO_3_^−^ at a concentration of 100 μg/ml dissolved in absolute DMSO, resulting in ~20 to 80 of viral RNA copies injected. Postinjection, the larvae were transferred to six-well plates containing Danieau’s solution and maintained in an incubator set to a 14-/10-hour light/dark cycle at 32°C. For baloxavir treatment experiments, baloxavir acid (MedChemExpress) was dissolved in absolute DMSO and added to the swimming water at a final concentration of 25 μM as described in (*53*), immediately after IAV H1N1 injection.

### RNA extraction and viral RNA quantification from zebrafish larvae

Each day postinjection, 10 zebrafish larvae were collected into 2-ml tubes containing 1.4-mm ceramic beads (Omni International) and immediately stored at −80°C for subsequent analysis. For RNA extraction, 350 μl of TRIzol reagent (Thermo Fisher Scientific) was added to the frozen larvae within the microtubes. The samples were homogenized for 10 s at 6300 rpm using a homogenizer (Bertin Technologies). After homogenization, the lysate was clarified by centrifugation at 10,000*g* for 2 min, and the supernatant was carefully transferred to a sterile Eppendorf tube. An equal volume of absolute ethanol was mixed with the supernatant, and viral RNA was extracted using the Direct-zol RNA Miniprep Kit (Zymo Research), following the manufacturer’s instructions. The purified RNA was stored at −80°C until further quantification by RT-qPCR. Detection of viral RNA was carried out using a one-step RT-qPCR protocol with the iTaq Universal Probes One-Step Kit (Bio-Rad). The primers used were 5′-GACCRATCCTGTCACCTCTGAC-3′ and 5′-AGGGCATTYTGGACAAAKCGTCTA-3′, along with the probe 5′-6-FAM/TGCAGTCCT/ZEN/CGCTCACTGGGCACG/IABkFQ-3′ (*84*). Amplification was performed on a QuantStudio 5 instrument (Thermo Fisher Scientific) with the following cycling conditions: reverse transcription at 50°C for 10 min, initial denaturation at 95°C for 2 min, and 40 cycles of 95°C for 15 s followed by 60°C for 30 s. For absolute quantification, standard curves were generated using 10-fold serial dilutions of a DNA template with a known concentration and the sequence 5′-CTAAAGACAAGACCAATCTTGTCACCTCTGACTAAGGGAATTTTAGGATTTGTGTTCACGCTCACCGTGCCCAGTGAGCGAGGACTGCAGCGTAGACGCTTTGTCCAAAATGCCCTAAATGGGAATGGGGACCC-3′.

### Statistics

In vitro experiments were performed at least in duplicate with at least two independent experiments. Ex vivo experiments were performed with at least two MucilAir in independent experiments. Results are shown as the means and SEM. EC_50_ and 50% cytotoxicity concentration were calculated with GraphPad Prism version 9.1 as described in Mathez and Cagno (*78*). Analysis of variance (ANOVA) followed by multiple comparison analysis, unpaired two-tailed *t* test, area under the curve followed by additional statistical test, and Mann-Whitney test were used using GraphPad Prism version 9.1. For each figure, the number of independent experiments and the statistical test used if applicable are written.

### Ethics

This study was carried out in accordance with INRAE guidelines in compliance with European animal welfare regulation. The protocols were approved by the Animal Care and Use Committee at “Centre de Recherche de Jouy-en-Josas” under relevant institutional authorization (“Ministère de l’éducation nationale, de l’enseignement supérieur et de la recherche”), authorization number: 2020010910262184 v4 (APAFIS#25172). Zebrafish experiments were approved and performed according to the rules and regulations of the Ethical Committee of KU Leuven (P070/2021), in compliance with the European Union regulations concerning the welfare of laboratory animals as declared in Directive 2010/63/EU.
